# Experimental demonstration of improved magnetorelaxometry imaging performance using optimized coil configurations

**DOI:** 10.1002/mp.15594

**Published:** 2022-03-17

**Authors:** Peter Schier, Maik Liebl, Uwe Steinhoff, Frank Wiekhorst, Daniel Baumgarten

**Affiliations:** ^1^ Institute of Electrical and Biomedical Engineering UMIT ‐ Private University for Health Sciences, Medical Informatics and Technology Hall in Tirol Austria; ^2^ Physikalisch‐Technische Bundesanstalt (PTB) Berlin Germany; ^3^ Institute of Biomedical Engineering and Informatics Technische Universität Ilmenau Ilmenau Germany

**Keywords:** condition number, imaging performance, magnetic nanoparticles, magnetorelaxometry imaging, optimization

## Abstract

**Background:**

Magnetorelaxometry imaging is an experimental imaging technique capable of reconstructing magnetic nanoparticle distributions inside a volume noninvasively and with high specificity. Thus, magnetorelaxometry imaging is a promising candidate for monitoring a number of therapeutical approaches that employ magnetic nanoparticles, such as magnetic drug targeting and magnetic hyperthermia, to guarantee their safety and efficacy. Prior to a potential clinical application of this imaging modality, it is necessary to optimize magnetorelaxometry imaging systems to produce reliable imaging results and to maximize the reconstruction accuracy of the magnetic nanoparticle distributions. Multiple optimization approaches were already applied throughout a number of simulation studies, all of which yielded increased imaging qualities compared to intuitively designed measurement setups.

**Purpose:**

None of these simulative approaches was conducted in practice such that it still remains unclear if the theoretical results are achievable in an experimental setting. In this study, we demonstrate the technical feasibility and the increased reconstruction accuracy of optimized coil configurations in two distinct magnetorelaxometry setups.

**Methods:**

The electromagnetic coil positions and radii of a cuboidal as well as a cylindrical magnetorelaxometry imaging setup are optimized by minimizing the system matrix condition numbers of their corresponding linear forward models. The optimized coil configurations are manufactured alongside with two regular coil grids. Magnetorelaxometry measurements of three cuboidal and four cylindrical magnetic nanoparticle phantoms are conducted, and the resulting reconstruction qualities of the optimized and the regular coil configurations are compared.

**Results:**

The computed condition numbers of the optimized coil configurations are approximately one order of magnitude lower compared to the regular coil grids. The reconstruction results show that for both setups, every phantom is recovered more accurately by the optimized coil configurations compared to the regular coil grids. Additionally, the optimized coil configurations yield better signal qualities.

**Conclusions:**

The presented experimental study provides a proof of the practicality and the efficacy of optimizing magnetorelaxometry imaging systems with respect to the condition numbers of their system matrices, previously only demonstrated in simulations. From the promising results of our study, we infer that the minimization of the system matrix condition number will also enable the practical optimization of other design parameters of magnetorelaxometry imaging setups (e.g., sensor configuration, coil currents, etc.) in order to improve the achievable reconstruction qualities even further, eventually paving the way towards clinical application of this imaging modality.

## 1 INTRODUCTION

Various novel therapeutic approaches rely on the injection of magnetic nanoparticles (MNPs) into the human body. Among others, these approaches include magnetic drug targeting^1^, ^2^ and magnetic hyperthermia,^3^, ^4^, ^5^ both of which are especially promising in the area of cancer treatment. Nevertheless, it is required to have a precise knowledge about the spatial in vivo MNP distribution prior to and during these treatments in order to ensure a safe and effective procedure. Additionally, quantitative information about the MNP concentration is also necessary since the number of particles in the region of interest (ROI) is directly related to the drug concentration during magnetic drug targeting and to the heat dissipation during magnetic hyperthermia in this area.

Established clinical imaging modalities are limited in their ability to accurately and quantitatively reconstruct MNP ensembles inside a volume. Although it is possible to detect and quantify MNPs by magnetic resonance imaging (MRI),^6^ there are several drawbacks to be considered. The reduced $T_{2}$ times of protons of tissue containing MNPs cause a strong negative contrast (“black spots”) in the MRI images which can be ambiguous and misinterpreted as hemorrhages or air bubbles (and vice versa)^7^. Furthermore, the accurate quantification of the MNPs by MRI additionally requires the knowledge of the respective tissue relaxation times without MNPs.^8^ This introduces further uncertainties to the quantification since these relaxation times can change over time. Paysen et al.^8^ demonstrated the feasibility of MNP quantification using an MRI scanner but reported a narrow range of MNP concentrations that can be visualized and quantified accurately. The MNP‐induced changes of the proton $T_{2}$ times were either undetectable or produced distorting imaging artifacts outside of this range.

Magnetic particle imaging (MPI)^9^, ^10^ is an emerging imaging modality that has been designed for quantitative reconstruction of MNP distributions by detecting their nonlinear dynamic response to a time‐varying magnetic drive field. MPI is a highly sensitive and MNP‐specific imaging technique with current detection limits down to about 1 ng of iron in a submillimeter spatial resolution^11^ and allows real‐time imaging in the millisecond regime.^12^, ^13^ Furthermore, the dynamic magnetic behavior of the MNPs can be used as a direct probe as it is very sensitive to the physiological environment, detected as changes of the MNPs' relaxation properties. The quantitative determination of the relaxation properties during this so‐called multicolor MPI allows for the separation of different MNP systems^14^ and yields environmental information.^15^ MPI shows huge potential to improve a number of MNP‐related biomedical applications such as cancer detection, neuro‐ and cardiovascular imaging, cell tracking, and magnetic hyperthermia.^16^ Nonetheless, for successful clinical translation of MPI, there are challenges to be addressed. One of the biggest is the upscaling of MPI scanners to fit a human body (part). Currently available scanners are designed with an ROI in the size of a rodent,^17^ although promising efforts towards imaging human body parts are currently pursued.^18^, ^19^ The enlargement of the ROI has shown to be demanding because of arising safety concerns such as tissue heating and peripheral nerve stimulation that need to be considered^16^, ^17^ and substantially increased hardware requirements.^18^, ^19^ These challenges are actively approached by developing low drive‐amplitude strategies^20^, ^21^ and high‐performance MNP systems.^22^, ^23^ Nonetheless, more research and technical advances will be necessary to develop a clinically applicable MPI scanner.

Magnetorelaxometry imaging (MRXI)^24^ is another powerful MNP imaging modality, which utilizes the response of the magnetic moments of MNPs to the rapid shut down of a magnetizing magnetic field. Similarly, MRXI is capable of noninvasive, quantitative, and highly specific reconstruction of MNP distributions.^25^ Although the spatial and temporal resolution as well as the MNP detection limits of MRXI (about 1 cm spatial resolution recorded in few seconds to minutes^25^, ^26^ down to about 10 ng of iron content^27^) lacks the capabilities of MPI for small ROIs (few cubic centimeters^13^), MRXI reconstructions of MNP distributions in medium‐sized ROIs^25^, ^26^, ^28^ are of similar quality and spatial resolution as those of recent experimental MPI scanners of this size.^19^ For instance, Jaufenthaler et al.^26^ demonstrated that recovering two MNP filled voxels with a side length of 12 mm, separated by a single voxel length, is unproblematic using a two‐dimensional MRXI setup with an ROI of $12\times 8\;{\mathrm{cm}}^{2}$. In another study,^28^ we showed that the reconstruction of different three‐dimensional (3D) MNP distributions inside an ROI of $12\times 12\times 6\;{\mathrm{cm}}^{3}$ within a phantom body of $50\times 40\times 7{\;\mathrm{cm}}^{3}$ works very well using the same voxel resolution, optimized MRXI coil currents, and appropriate regularization techniques. Given the quality of the reconstructions in these two studies, it is reasonable to assume that higher resolutions would have been achievable. Gräser et al.^19^ developed a human head‐sized MPI with a bore size of 19 cm × 25 cm and a measurable ROI of about $10\times 14\times 10\;{\mathrm{cm}}^{3}$ and demonstrated lower resolution limits of 5 , 6 , and 26 mm, depending on the spatial orientation of the MNP source separation phantom. Considering the largely still unoptimized design of the MRXI setups and its data acquisition procedure, the slight enlargement of the ROI and the (partly) lower resolution limits that are demonstrated by this MPI scanner are well within the capabilities of the MRXI modality. Furthermore, since MRXI applies static magnetic fields with smaller amplitudes than that of MPI, an upscaling of MRXI to enable scanning larger ROIs (e.g., a torso or an entire human body) is technically less challenging and implies no safety concerns. As such, MRXI has its benefits as another promising MNP imaging approach.

Image reconstruction in MRXI requires the solution of the ill‐posed inverse problem of the linear MRXI forward model. This ill‐posedness (combined with measurement noise and model inaccuracies) prevents a unique solution to the inverse problem and hampers accurate imaging results. The excitation coil configurations that generate the magnetic fields for MRXI have a major impact on the mathematical composition of the system matrix and thus also on the ill‐posedness and the quality of the reconstructions. Multiple simulation studies were conducted to optimize the applied magnetic field configurations during MRXI, thereby mitigating the ill‐posedness and promoting more accurate and high‐contrast reconstruction results. These studies were involved in the optimization of the excitation coil currents^28^, ^29^, ^30^, ^31^ and the excitation coil configurations.^32^, ^33^ Although the synthetic data presented in these studies show improved imaging performances as a result of the proposed optimizations, the efficacy and feasibility of the approaches above have not been validated in the practice.

Therefore, the goal of this study is to calculate optimized MRXI setups and to experimentally validate their improved imaging quality compared to previous intuitively designed MRXI setups. To date, the best practice in experimental MRXI setup design is to use regularly arranged excitation coil grids closely covering the outer surface of the ROI^25^, ^26^ or to use huge Helmholtz coils^24^, ^34^ for magnetizing the MNP ensembles and to measure their response with a regular arrangement of superconducting quantum interference devices (SQUIDs)^24^, ^25^, ^34^ or optically pumped magnetometers (OPMs).^26^ Obviously, these intuitive setup designs are not optimized and often spatially encode parts of the ROI redundantly or not sufficiently. Such optimization is mandatory for the advance of MRXI to increase the reliability and the accuracy of this imaging modality to an extent where its clinical application becomes feasible. By providing this proof of concept, we take the first step towards practical MRXI setup optimization.

In our earlier work, we conducted an extensive simulation study in which we constructed a routine for the global optimization of the excitation coil configurations for two distinct MRXI setups.^35^ The experiments in the present study shall be based upon the cuboidal and cylindrical ROIs investigated in our earlier work. We will determine initial coil configurations for both ROIs by means of the presented discrete global optimization approach.^35^ The initial coil configurations shall then be continuously optimized with a subsequent constrained local optimization approach allowing for the design of technically feasible coil configurations. Additionally, regular coil configurations will be used for the imaging of both ROIs. The achievable reconstruction accuracies of the manufactured regular and optimized coil configurations are compared with MRXI measurements of several different technologically and clinically relevant spatial MNP distributions (or *phantoms*). The comparison of the resulting imaging qualities shall prove that theoretically optimized MRXI designs can indeed be transferred into practical setups and deliver more accurate reconstructions of the MNP distributions than intuitively designed MRXI systems.

## 2 MATERIALS AND METHODS

### 2.1 Magnetorelaxometry imaging

The principle of MRXI relies on a two‐step procedure where an ROI containing MNPs is initially magnetized for a certain time using an external magnetic field generated by $N_{c}$ electromagnetic coils to align the magnetic moments of the superparamagnetic cores of the particles. Subsequently, after switching off the magnetizing field, the relaxing net magnetic moment is recorded by $N_{s}$ highly magnetosensitive sensors. Typically, this two‐step procedure is repeated $N_{a}>1$ times with different external magnetic field configurations for proper spatial encoding of the ROI that enables a more accurate reconstruction of the MNP distribution inside it. The ROI is virtually tessellated into $N_{v}$ equally sized volume elements (voxels) to formulate a discrete forward model of the MRXI procedure that is used to recover the locations and quantities of the MNPs from the solution of the corresponding inverse problem.

The forward model of MRXI can be described as a system of linear equations and is formulated by

(1) $$\begin{array}{c} Lx=b, \end{array}$$

where $L\in R^{N_{s}N_{a}\times N_{v}}$ denotes the system matrix, $x\in R^{N_{v}}$ the vector of the unknown MNP core masses inside the $N_{v}$ voxels, and $b\in R^{N_{s}N_{a}}$ the measurement vector holding the relaxation amplitudes that are recorded by $N_{s}$ sensors throughout $N_{a}$ activation sequences. The system matrix L encodes the geometrical and (electro)magnetic interrelationships between the coils, the sensors, the voxels, as well as the a priori known magnetic properties of the MNPs. The complete system matrix L is composed of the system matrices from the individual activation sequences $L_{a}\in R^{N_{s}\times N_{v}}$ that are concatenated below each other such that

(2) $$\begin{array}{c} L={\left[\begin{array}{ccccc} L_{1}^{T} & \cdots & L_{a}^{T} & \cdots & L_{N_{a}}^{T} \end{array}\right]}^{T}. \end{array}$$

We use an established choice for the magnetic field configurations where a different coil is driven by a uniform coil current during each activation sequence such that $N_{a}=N_{c}$.^25^, ^36^

The solution of the ill‐posed inverse problem of the forward model (1) requires the application of a regularization technique, enabling the reconstruction of physically reasonable estimates of the true particle ensembles based on the introduction of additional penalty functions (i.e., the *regularizers*). We employ the two different, sensitivity‐weighted regularization methods^28^ adapted to MRXI, which are based on an iterative Tikhonov regularization^37^ and an iterative shrinkage‐thresholding algorithm,^38^ respectively. The sensitivity‐weighted Tikhonov regularization, henceforth referred to as SWTikh, promotes smooth estimates of the MNP distributions and penalizes outliers. The sensitivity‐weighted iterative shrinkage‐thresholding algorithm, henceforth referred to as SWISTA, promotes sparse solutions with few nonzero elements in x. These two different regularization methods are applied for the reconstruction of the MNP distributions to show the efficacy of the coil configuration optimization across different solution approaches.

**FIGURE 1:**
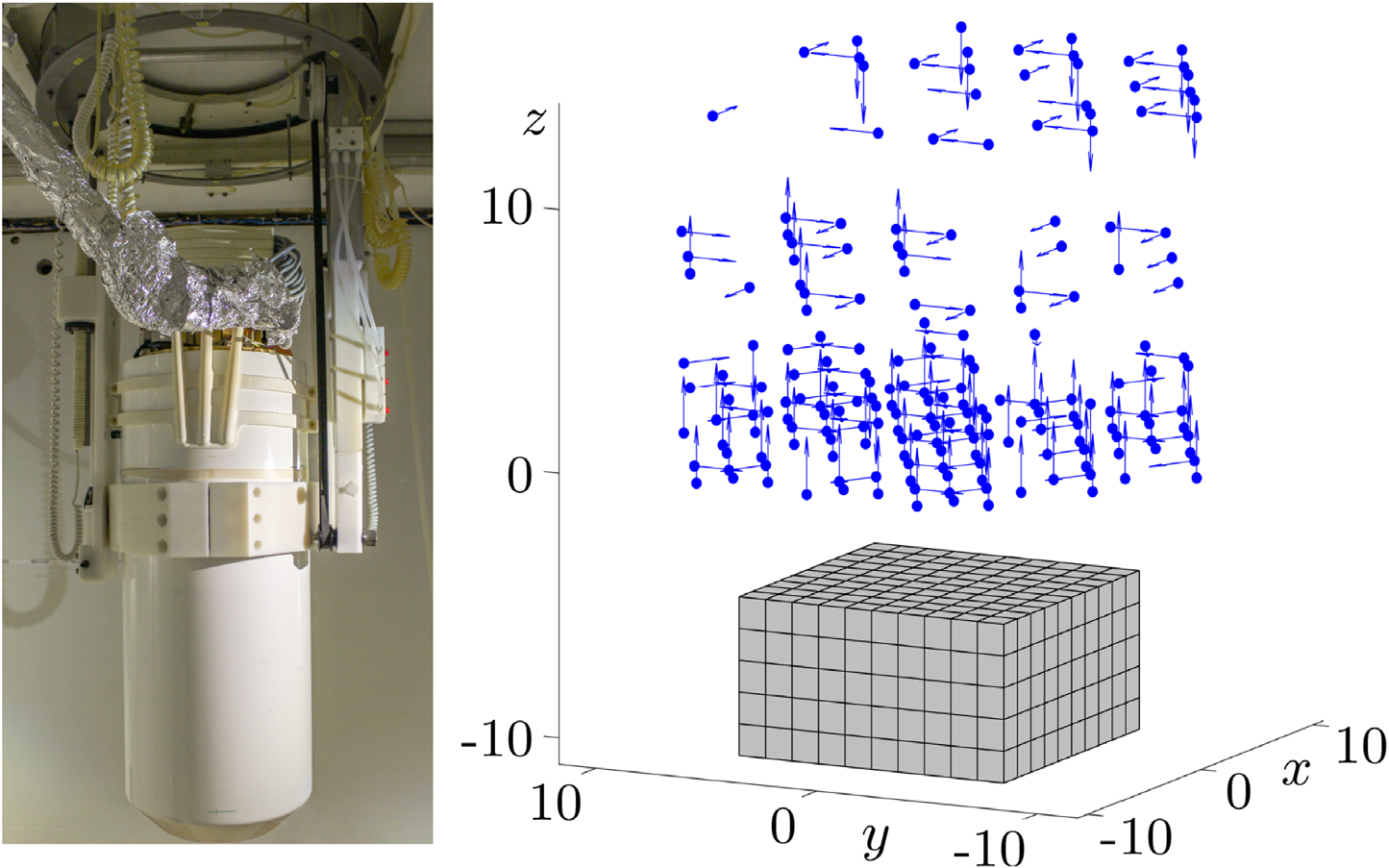
The photograph shows the 304 SQUID vector magnetometer system of PTB Berlin.^39^ The sensors are encased in the bottom half of the cylindrical dewar that is required for the liquid helium cooling of the SQUIDs. The right image shows a representation of the $N_{s}=194$ sensor positions and their respective sensitive axes in blue. The cuboidal ROI, as employed in the practical experiments and tessellated into 10×10×5 voxels, is depicted below the sensor system in gray. The axis dimensions are given in centimeters.

We utilized the 304 SQUID vector magnetometer system from the Physikalisch‐Technische Bundesanstalt (PTB)^39^ for recording the relaxation signals, whereas only $N_{s}=194$ sensors were active at the time the experiments were conducted. The SQUID sensors are hexagonally arranged on four horizontal layers with outer diameters of approximately 20 cm, and their sensitive axes are oriented in five different directions. The real sensor system and a virtual representation thereof are depicted in Figure 1.

### 2.2 Optimization of MRXI coil configurations

We optimized the coil positions and radii of a cuboidal as well as a cylindrical coil configuration by minimizing the condition numbers of their respective system matrices L. The condition number is calculated by

(3) $$\begin{array}{c} \kappa \left(L\right)={\left\|L\right\|}_{2}{\left\|L^{+}\right\|}_{2}, \end{array}$$

where ${∥\cdot ∥}_{2}$ represents the spectral matrix norm and $L^{+}$ denotes the Moore–Penrose inverse of the system matrix. The condition number is an indicator for the stability of linear inverse problems^40^, ^41^ and it was shown in a simulation study that the minimization of κ(L) is an effective approach to optimize the reconstruction quality of MRXI setups.^35^

Typically, κ(L) exhibits a vast amount of local minima when the excitation coil positions and radii are varied. Thus, the optimization was conducted in two consecutive steps to determine an objective function value close to the global minimum of κ(L). Initially, a large number of coils with different positions and radii were placed around the cuboidal as well as the cylindrical ROIs in our MRXI simulation environment. The first part of the optimization process involved the selection of a subset of $N_{c}=32$ coils with minimal κ(L) out of these large coil configurations by means of global tabu search optimization.^35^ According to the results shown in this study, a coil count of $N_{c}=32$ is suitable for ROIs of this size and the PTB sensor system.

This preliminary global optimization was performed to obtain a good initialization of the coil positions and radii for the second part of the optimization process. The coil configurations were refined by means of a local quasi‐Newton optimization, further minimizing κ(L) in this second step. The positioning of the coils was constrained during this step of the optimization to generate practically manufacturable coil configurations. For the cuboidal coil configuration, the coils were constrained to four planes that lay in parallel to the faces of the ROI in negative and positive y‐ and z‐directions, each with a distance of 3 mm to the ROI. The coils for the cylindrical configuration were positioned on a tubular constraint around the ROI with the shortest distance of 1 cm between the constraint and the ROI. The resulting coil configurations are explained in detail in Section 2.3.

### 2.3 Employed MRXI setups

#### 2.3.1 Cuboidal setups

**FIGURE 2:**
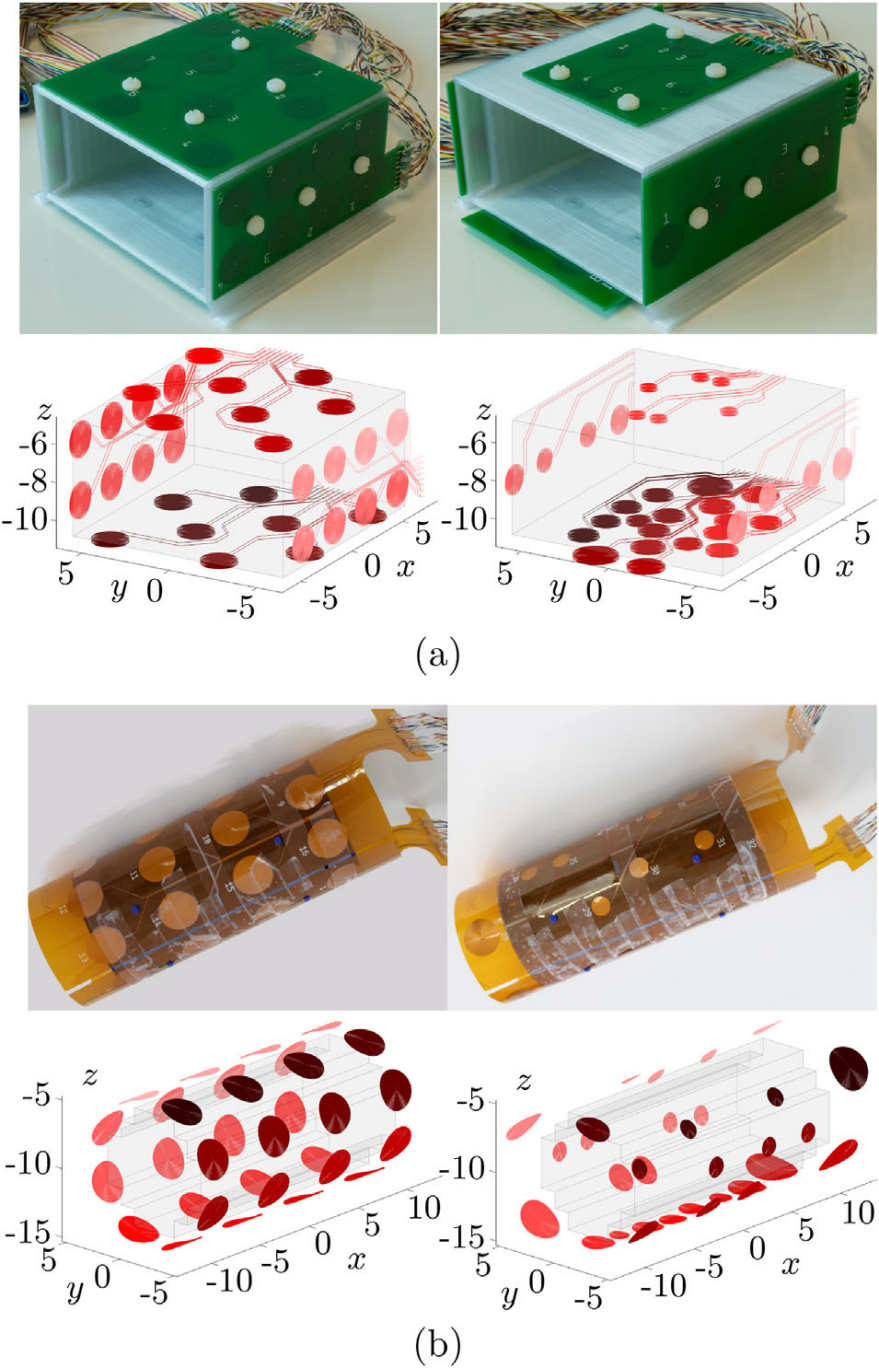
Regular (left) and optimized (right) cuboidal (a) and cylindrical (b) MRXI coil configurations. The top rows depict the real setups used for MRXI measurements. The bottom rows show the corresponding representations of the coils (red) and the ROIs (light gray) in the MRXI simulation environment used for calculating the forward model and solving the inverse problem. The different shades of red illustrate the sequences of the coil activations from first activations (dark) to last activations (light). Axis dimensions are given in centimeters.

Two coil configurations for MRXI measurements of the cuboidal ROI were realized and used in practice: a regular coil configuration and the configuration with coil positions and radii optimized using the approach presented in section 2.2. The practical realization of the two coil configurations as well as their virtual representations in the MRXI simulation environment is depicted in Figure 2a. The regular coil configuration was designed intuitively with 8 coils uniformly distributed over each of the four available faces of the ROI, similar to the configurations employed in another experimental MRXI study.^36^

The coils for both configurations are printed on four double‐layer printed circuit boards (PCBs) with a thickness of 1.6 mm, respectively. Every coil exhibits a spiral wire pattern with a wire thickness of 0.25 mm and a wire‐to‐wire distance of 0.4 mm. The coils are printed on both sides of the PCBs and are interconnected through the center of the spirals to increase the number of windings and hence the technically feasible magnetic field amplitudes. The incoming and outgoing connection traces from each coil lie parallel to each other on top and the bottom of the PCBs and are printed from the outermost points of the coils to the edges of the PCBs, where they have been connected to twisted‐pair cables leading to the control unit that governs the activation sequences. The wiring was designed in such a way to minimize stray magnetic fields from the connection lines.

The PCBs were mounted on a 3D printed cuboidal phantom holder made from polylactic acid (PLA) and were fixated using plastic screws. The wall thickness of the phantom holder (i.e. the distance between PCBs and ROI) is approximately 3 mm. The cuboidal setups were placed centrally beneath the sensor system with a vertical distance of roughly 5 cm between the lowest sensor plane and the topmost PCB.

#### 2.3.2 Cylindrical setups

Analogous to the cuboidal setups, a regular coil configuration as well as an optimized coil configuration was constructed for MRXI measurements of a cylindrical ROI. The two real coil configurations as well as their virtual versions required for the solution of the MRXI inverse problem are depicted in Figure 2b. The regular coil configuration was designed as regular coil grid with four rings of coils around the cylindrical ROI, each of which employs eight regularly spaced coils, respectively.

The coil configurations are realized as flexible printed circuits (FPCs), allowing a pliable bending of the coil configurations and thus a tight fit of the FCPs around the tubular phantom container that encapsulates the cylindrical ROI. Except for the thickness of the FPCs which is 0.2 mm, the geometrical parameters of the coil configurations and the design of the printed coils are identical to those of the cuboidal coil configurations.

The tubular phantom holder was 3D printed from PLA as well. The FPCs were manufactured with bores at defined positions which were attached to 3D printed pins on the surface of the phantom holder (see the small blue protrusions in the top panels of Figure 2b) to guarantee a defined positioning of the coil configurations. Additionally, the FPCs were affixed with adhesive tape. The shortest distance between the FPCs and the cylinder mantle of the ROI is 1 cm. The cylindrical setups were positioned centrally beneath the sensor system with a vertical distance of roughly 5 cm between the lowest sensor plane and the topmost section of the FPCs' cylindrical mantle.

### 2.4 Employed MNP phantoms

Multiple MNP phantoms were created to assess the reconstruction qualities of the various coil configurations. These 3D MNP phantoms were constructed by arranging several gypsum cubes with embedded MNPs into different shapes as it was done before by Liebl et al.^25^ This immobilization impedes the Brownian motion^42^ of the MNPs and permits exclusively Néel relaxation^43^ of magnetic moments of the MNPs. Since the imaging performance of different coil configurations does not rely on any specific peculiarities regarding the underlying relaxation mechanisms of the MNP, we exclude any changes in the relaxation curve by using immobilized MNP on purpose. Every gypsum cube has an edge length of 1.2 cm and contains an MNP iron mass of 6.37 mg. The MNPs were manufactured by Berlin Heart GmbH, Germany, with a mean core diameter of 20 nm^26^ and exhibit an iron concentration of $c\left(\mathrm{Fe}\right)=0.9\;{\mathrm{mmol}\;L}^{-1}$.^36^

**FIGURE 3:**
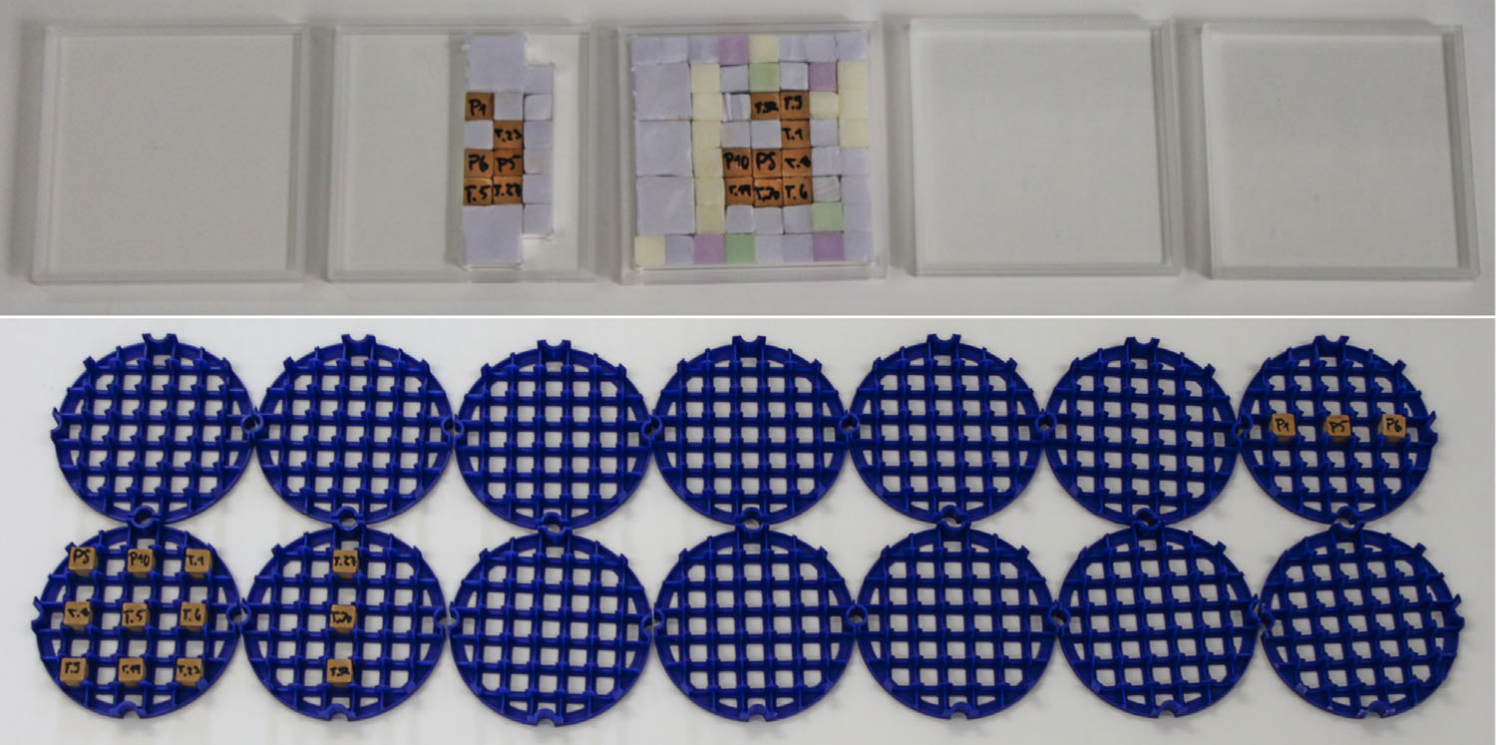
Examples of MNP gypsum cube arrangements for assembling the 3D phantoms. The top photograph shows the disassembled Tumor phantom for the cuboidal ROI, and the bottom photograph shows the disassembled Separation phantom for the cylindrical ROI. The frames/disks were stacked upon each other, beginning from (top) left to (bottom) right.

The cubes were placed within five separate polymethylmethacrylate (PMMA, Plexiglas^®^) frames and stacked upon each other to assemble the 3D MNP phantoms for the cuboidal ROI. Similarly, the cubes were inserted into 3D printed circular disks made from PLA and are successively inserted into the tubular phantom holder to create the cylindrical MNP phantoms. Examples of disassembled cuboidal and cylindrical MNP phantoms are depicted in Figure 3.

**FIGURE 4:**
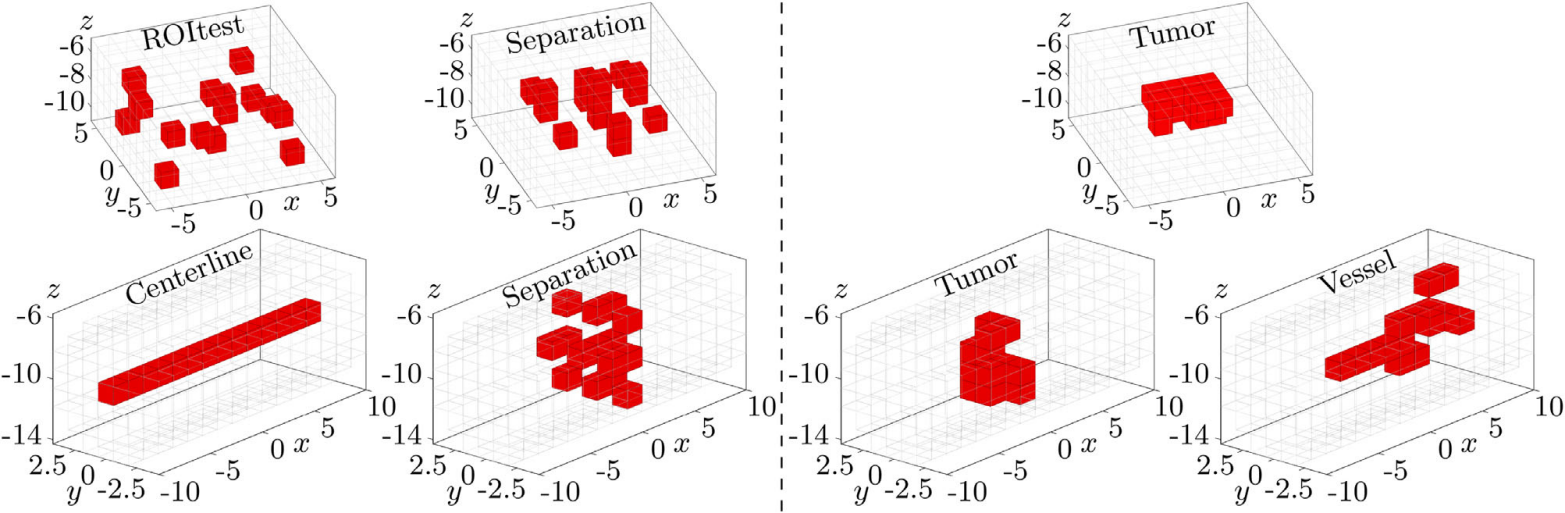
Cuboidal (top) and cylindrical (bottom) MNP phantoms applied for MRXI measurements. The technical phantoms are depicted on the left side and the anatomical phantoms on the right side. Each red voxel in the phantoms represents one MNP infused gypsum cube with an MNP iron mass of approximately 6.37 mg. Axis dimensions are given in centimeters.

Seven MNP phantoms were built this way, three for the cuboidal ROI and four for the cylindrical ROI. Virtual representations of all phantoms are shown in Figure 4. The phantoms are subdivided into anatomical phantoms that replicate relevant anatomical structures containing MNPs and technical phantoms that shall test the imaging capabilities of the different MRXI setups. The anatomical phantoms Tumor and Vessel are designed to resemble tumor‐like and vascular structures, respectively. The purpose of the difficult to reconstruct technical Separation phantoms is to provide information about the resolution and separation capabilities of the coil configurations. The technical phantoms ROItest and Centerline shall demonstrate the feasible reconstruction accuracies in different areas of the cuboidal ROI and test the imaging qualities in lowly sensitive areas deep inside the cylindrical ROI, respectively.

### 2.5 Measurement procedure, preprocessing, and reconstructions

MRXI measurements were performed in the magnetically shielded room BMSR‐2 at PTB using their MRXI equipment (coil current control unit, sensor data recording system, etc.), the four coil configurations, and all of the respective phantoms. Every measurement was conducted according to the following procedure. All 32 electromagnetic coils were individually and sequentially energized with approximately 1 A using the cuboidal and 0.7 A using the cylindrical coil configurations for a duration of 1 s. The driving coil current was switched off, and the resulting relaxation signals were recorded for 2 s with a sampling rate of 1000 Hz, 150 μs (required for SQUID recovery) after each coil activation. Each measurement was repeated five times to compensate for faulty recordings and to enable a subsequent automated rejection of recordings with poor repeatability, resulting in a certain amount of usable relaxation amplitudes $N_{\mathrm{usable}}\leq N_{s}N_{c}$ for the reconstruction of the different phantoms.

Additionally, empty measurements without MNP phantoms inside the MRXI setups were performed using the previously described measurement procedure to record spurious signals from eddy currents in the environment. These empty measurements were subtracted from the recorded relaxation signals of each of the corresponding measured phantoms, resulting in cleaned relaxation signals only accounting for the decaying net magnetization of the MNPs.

A sum of four exponential functions

(4) $$\begin{array}{c} b_{\mathrm{fit}}\left(t\right)=A_{\mathrm{Offset}}+\sum_{j=1}^{4}A_{j}\cdot \exp\left(-\frac{t}{\tau_{j}}\right) \end{array}$$

was fitted to every cleaned relaxation signal to denoize the measurement data with $A_{\mathrm{Offset}}$, $A_{1\cdots 4}$, and $\tau_{1\cdots 4}$ denoting the fitting parameters. Already four exponential functions yielded good fit results for the vast majority of the recorded data. Subsequently, the relaxation amplitudes b were extracted from the fits by subtracting the fit function values at the two fixed time instances after the beginning of the relaxation processes $t_{1}=0.1\;s$ and $t_{2}=1.1\;s$ such that $b=b_{\mathrm{fit}}\left(t_{1}\right)-b_{\mathrm{fit}}\left(t_{2}\right)$. These relaxation amplitudes were averaged over the five repeated measurements.

Prior to the reconstructions, it was necessary to correctly localize the coil configurations relative to the sensor system. The coils were individually driven by sinusoidal currents with a frequency of 28.8 Hz and an amplitude of 20 μA for this purpose. Afterwards, the position and orientation of the sensor system in the simulation environment were varied by means of a quasi‐Newton optimization, thereby minimizing the root‐mean‐square deviation between the measured and the simulated localization amplitudes.

A reference measurement was conducted to enable a quantification of the MNP iron masses in the MRXI reconstructions. For this purpose, one gypsum cube with embedded MNPs (with known MNP iron mass) was placed at a defined location inside the optimized cuboidal coil configuration. The ratio between the simulated and the recorded relaxation amplitudes of the reference measurement yields a scaling factor that allows for accurate quantification of the MNP iron masses inside all phantoms when multiplied to their respective reconstructions. The interested reader is referred to a study by Wiekhorst et al.^27^ for a more detailed description of reference measurements and the scaling procedure.

**FIGURE 5:**
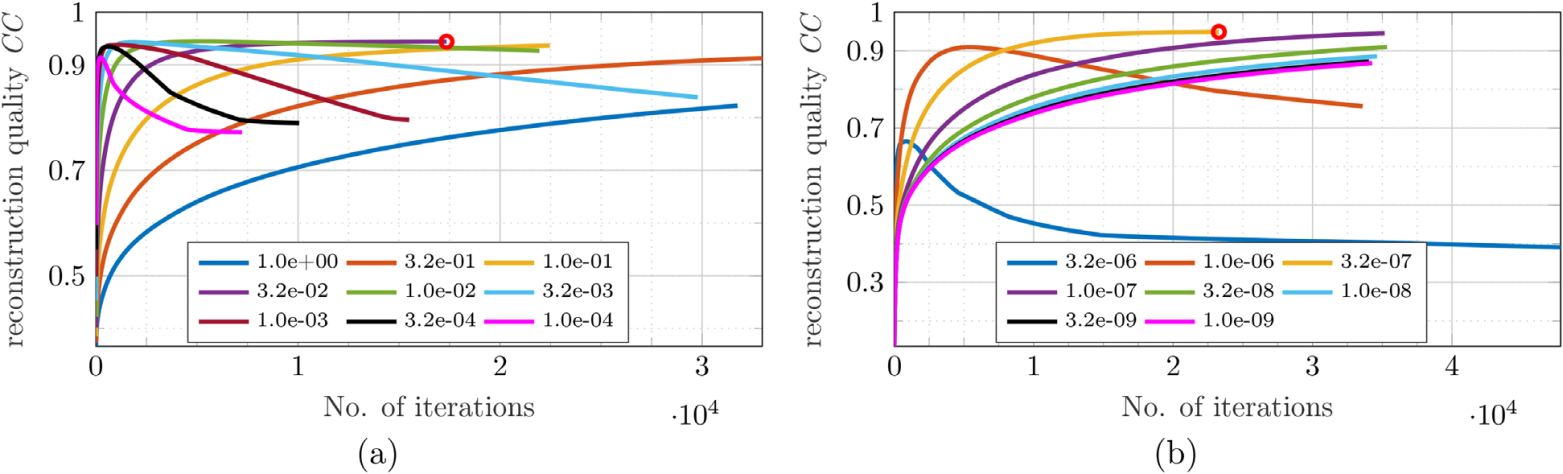
Dependency of reconstruction quality CC on the number of iterations of (a) SWTikh and (b) SWISTA regularization of the cylindrical Vessel phantom using different regularization parameters (see legends). The red circles indicate the terminated reconstructions with the largest achievable CC values that are used for evaluating the reconstruction performances. The regularization parameter value $10^{-5}$ for SWISTA was too large for this reconstruction and yielded no meaningful result.

Every phantom measured with the regular and the optimized coil configurations of the cuboidal and the cylindrical ROIs was reconstructed using both the SWTikh as well as the SWISTA algorithms (see Section 2.1). The termination criterion for both regularization algorithms was set to a minimum deviation between current and previous iteration step $∥x_{j}-x_{j-1}∥/∥x_{j-1}∥$ of 0.1%. The reconstruction quality is measured by the Pearson correlation coefficient *CC* between the true MNP phantom $x_{\mathrm{true}}$ and its reconstruction $x_{j}$ such that

(5) $$\begin{array}{c} CC=\frac{\mathrm{cov}(x_{\mathrm{true}},\;x_{j})}{\sigma_{\mathrm{true}}\;\sigma_{j}} \end{array}$$

with cov(·,·) denoting the covariance between the two MNP distributions and σ their respective standard deviations. The CC quantifies the similarity of the two images and ranges from −1 (perfect anticorrelation) over 0 (no correlation) to 1 (perfect reconstruction). Every regularization was repeated using different regularization parameters between $10^{-4}$ and 1 for SWTikh and between $10^{-9}$ and $10^{-5}$ for SWISTA in nine logarithmically equidistant steps, respectively. The reconstruction that terminated with the largest achievable CC value has been stored for the evaluation of the imaging performances for each phantom, every setup, and both regularization approaches. An illustration of this procedure and the different convergence properties is depicted in Figure 5.

Finally, the signal fidelities of the different phantom measurements are assessed by the model deviation D. Here, D is given in dB and quantifies the similarity between the measured relaxation amplitudes $b_{\mathrm{meas}}$ and the simulated amplitudes $b_{\mathrm{sim}}$ of the respective MNP phantoms with

(6) $$\begin{array}{c} D=20\cdot \log\left(\frac{{\left\|b_{\mathrm{meas}}\right\|}_{2}}{{\left\|b_{\mathrm{meas}}-b_{\mathrm{sim}}\right\|}_{2}}\right). \end{array}$$

## 3 RESULTS

### 3.1 Measurement properties

The resulting system matrix condition numbers of the regular (“reg”) and the optimized (“opt”) cuboidal and cylindrical setups are shown in Table 1. The condition numbers of the optimized setups are approximately one order of magnitude smaller compared to the regular setups. Note that a comparison between the cuboidal and the cylindrical configurations is not sensible in this respect since κ(L) can only be compared meaningfully between identical matrix sizes.

**TABLE 1:** Condition numbers κ(L) of employed MRXI setups

	κ(L)	
Setup	reg	opt
Cuboid	$6.8\times 10^{5}$	$6.2\times 10^{4}$
Cylinder	$5.2\times 10^{6}$	$5.2\times 10^{5}$

Note: “reg” refers to regular and “opt” refers to optimized.

**FIGURE 6:**
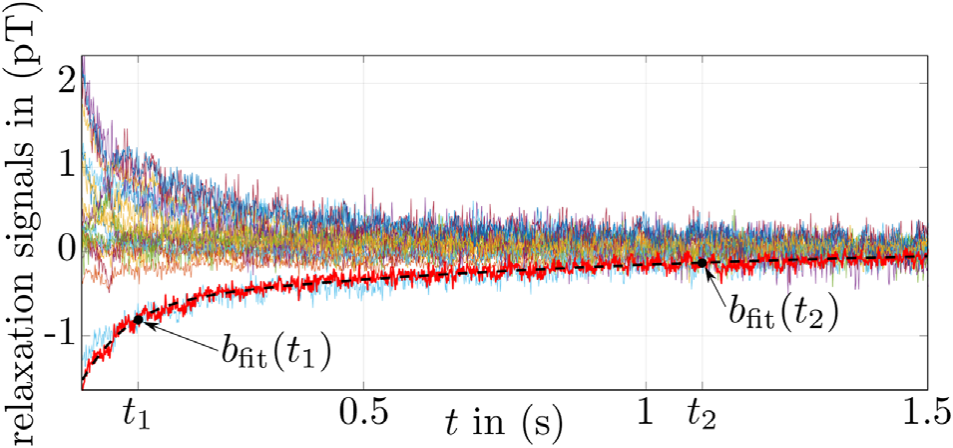
Examples of cleaned relaxation signals of an MRXI measurement. The black dashed line illustrates the fit result of the thick red signal including the two fit function values used to determine the relaxation amplitude

Figure 6 depicts signals from the different stages of the preprocessing routine employed to extract the relaxation amplitudes from the MRXI recordings (see Section 2.5). The described procedure was conducted for every recorded relaxation signal and yielded a certain number of usable relaxation amplitudes $N_{\mathrm{usable}}$ for each MRXI measurement.

### 3.2 Reconstruction performances

The maximally achievable CC values for regular and optimized coil configurations of the phantoms measured inside the cuboid‐ and cylinder‐shaped ROIs are displayed in Table 2. The best reconstruction performance of each phantom is marked in bold lettering. It is evident that the most accurate reconstruction of every phantom is provided by the optimized coil configurations, either by SWISTA or by SWTikh regularization. Additionally, a direct comparison of all CC values of a single regularization method shows that the optimized coil configurations outperform their regular counterparts in terms of reconstruction accuracy in every case, both for the SWISTA and the SWTikh methods.

**TABLE 2:** Reconstruction performances and signal quality measures

		CCSWISTA		CCSWTikh		D(dB)		$N_{\mathrm{usable}}$	
	Phantom	reg	opt	reg	opt	reg	opt	reg	opt
Cuboid	ROItest	0.90	**0.98**	0.92	**0.98**	16.0	16.1	5366	5178
	Separation	0.87	0.91	0.90	**0.95**	17.8	14.7	5457	5541
	Tumor	0.90	**0.98**	0.92	0.92	17.5	19.7	5343	5119
Cylinder	Centerline	0.98	**1.00**	0.96	0.97	13.8	16.0	4783	5066
	Separation	0.56	0.78	0.51	**0.85**	11.7	13.4	4530	4798
	Tumor	0.86	**0.91**	0.84	0.88	11.1	17.7	4620	4728
	Vessel	0.92	**0.95**	0.84	0.94	12.8	18.1	4609	5141

Note: “reg” refers to regular and “opt” refers to optimized. The bold numbers indicate the best performances per phantom.

The assessment parameters for the signal quality of the measured relaxation amplitudes $N_{\mathrm{usable}}$ and the signal fidelity measure D are presented in addition to the reconstruction quality measure CC in Table 2. The comparison of these two assessment parameters between regular and optimized coil configurations shows that almost every optimized configuration yields increased D values and a larger amount of usable measurements $N_{\mathrm{usable}}$.

**FIGURE 7:**
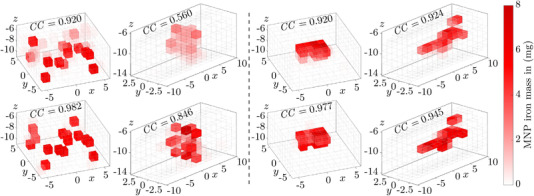
Examples of reconstructions with maximum attainable CC values (see Table 2) of regular coil configurations (top row) and optimized configurations (bottom row). Recovered MNP iron masses are shown in different shades of red. Additionally, the opacity of the voxels scales linearly with the contained MNP iron masses to increase visibility. The left half shows the technical cuboidal and cylindrical phantoms ROItest and Separation and the right half the anatomical phantoms Tumor and Vessel, respectively. Axis dimensions are given in centimeters.

Two reconstruction results of every employed coil configuration are depicted in Figure 7, displaying the reconstructions using the regular coil configurations in the top row and their optimized counterparts in the bottom row. The left half shows the reconstructions with the highest CC values of the technical cuboidal and cylindrical phantoms ROItest and Separation of both coil configurations, respectively. The visual comparison between the imaging results highlights the differences between the relatively inexact reconstructions of the regular coil configurations and the far more precisely recovered MNP distributions of the optimized coil configurations. Analogously, the right half of Figure 7 depicts the reconstructions of the anatomical phantoms Tumor and Vessel. Again, the top row displays blurrier, less precise reconstructions of the regular coil configurations and the bottom row more accurate MNP distributions recovered by the optimized coil configurations.

### 3.3 Fair comparison of reconstruction performances

Since the majority of relaxation amplitudes recorded with the optimized coil configurations exhibited increased D values and larger numbers of usable measurements $N_{\mathrm{usable}}$ compared to the regular setups, it is not yet certain that the better reconstruction accuracy can be fully attributed to the lower condition number of the system matrices. Therefore, we conduct an adjustment of the measurement data qualities to facilitate a fair comparison of the reconstruction performances.

The adjustment of the measurement data is performed by removing a number of data points to reach similar D values and identical $N_{\mathrm{usable}}$ values between the regular and the optimized measurements of every phantom, respectively. For each phantom, the worst data points with the largest deviations between measured and simulated relaxation amplitudes $|b_{\mathrm{meas}}-b_{\mathrm{sim}}|$ are removed from the sensor data with the lower D values until the higher D values are nearly equated. Subsequently, data points around the median deviation between measured and simulated relaxation amplitudes are removed from the measurements with larger $N_{\mathrm{usable}}$ values until an identical number of usable measurements is reached, thereby approximately maintaining the current D values.

The resulting assessment parameters of the signal quality after this adjustment and the maximally achievable CC values of the corresponding reconstruction results are shown in Table 3. The comparison of Tables 2 and 3 shows that the adjustment of the measurement data impacted the reconstruction accuracies only marginally. Mostly the CC values of reconstructions using the regular coil configurations increased slightly, which was expected since the majority of their measurement data adjustments resulted in larger D values. The reconstruction accuracies of the optimized coil configurations have not changed considerably. Nonetheless, the optimized coil configurations yield superior imaging qualities compared to the regular coil grids in every case.

**TABLE 3:** Reconstruction performances and signal quality measures after adjustment of measurement data

		CCSWISTA		CCSWTikh		rel. deviation		D(dB)	$N_{\mathrm{usable}}$
	Phantom	reg	opt	reg	opt	reg	opt	Both	Both
Cuboid	ROItest	0.89	**0.98**	0.90	**0.98**	19(8)	**6(3)**	16.1	5178
	Separation	0.87	0.91	0.90	**0.94**	**5(1)**	5(2)	17.8	5457
	Tumor	0.90	**0.98**	0.93	0.92	**3(1)**	8(4)	19.6	5000
Cylinder	Centerline	0.98	**1.00**	0.97	0.96	14(11)	**5(3)**	16.1	4600
	Separation	0.57	0.74	0.53	**0.83**	10(6)	**7(7)**	13.5	4400
	Tumor	0.88	**0.92**	0.86	0.87	10(4)	**5(2)**	17.9	4000
	Vessel	0.93	**0.94**	0.89	**0.94**	10(6)	**6(2)**	18.9	4000

Note: “reg” refers to regular and “opt” refers to optimized. The bold numbers indicate the best performances per phantom. The column “rel. deviation” represents the means and standard deviations (in parentheses) of the relative absolute quantification deviations between reconstructions and the magnetic nanoparticle iron masses employed in reality.

Additionally, the precision of the MNP iron mass quantification has been evaluated. The column “rel. deviation” in Table 3 represents the means and standard deviations (in parentheses) of the relative absolute quantification deviations between reconstructions and the MNP iron masses employed in reality. The optimized coil configurations quantify the underlying phantoms with similar or increased precision compared to the regular configurations. Only the cuboidal Tumor phantom is quantified more precisely using the regular coil grid. The MNP amounts recovered by the cuboidal regular coil configuration exhibit an average quantification uncertainty of 8.8±8.6% with respect to the practically employed MNP iron masses across all reconstructions with maximum CC values. Comparatively, the quantification uncertainty of cuboidal optimized coil configurations lies at 6.4±3.2%. The identical evaluation for the cylindrical coil configurations yields average quantification uncertainties of 10.9±7.2% and 5.7±4.1% for the regular and the optimized setups, respectively. Hence, on average, both optimized coil configurations are able to quantify the employed MNP iron masses more precisely than their regular counterparts.

## 4 DISCUSSION

The goal of this study is to experimentally validate the improved MRXI reconstruction accuracies of coil configurations optimized with respect to κ(L) over intuitive coil grid designs as suggested in our previous simulation study.^35^ For this purpose, an optimized as well as a regular coil configuration were manufactured to perform MRXI on two different ROIs, respectively (see Figure 2). The regular coil configurations were designed such that the ROIs are uniformly surrounded by equally sized coils in every direction. The optimized coil configurations employ a larger number of coils below the ROIs and the number of coils decreases towards the tops of the ROIs. This is related to the fact that the spatial separability of the voxels declines with increasing distance to the coils and sensors. Since the tops of the ROIs are closer to the sensors, the spatial separability of the lower parts has to be compensated by a larger amount of coils. Similarly, the expected relaxation signal strength of MNPs in a voxel also depends on the distances to coils and sensors. Therefore, the lower voxels have to be exposed to larger magnetic fields (thus the larger coil radii on the bottom) to make up for the larger distances to the sensors.

Three MNP phantoms were imaged in the cuboidal ROI using the regular and the optimized coil configurations, both of which were printed on stiff PCBs. Analogously, four different MNP phantoms in the cylindrical ROI were reconstructed with both coil configurations printed on pliable FPCs. The second anatomical phantom Vessel was only imaged in the cylindrical ROI due to the oblong shapes of both phantom and ROI.

The phantoms were reconstructed using the sensitivity‐weighted regularization methods SWTikh and SWISTA that were adapted to increase imaging performance in MRXI.^28^ The reconstructions with the largest achievable CC values of every setup and every employed phantom are used for further evaluation to enable an objective comparison of the imaging performances of the different coil configurations.

Since the PCB thickness of 1.6 mm is a relatively large distance between the connection traces, their contributions to the resulting magnetic fields of the cuboidal coil configurations are not negligible. Their incoming and outgoing connection traces are considered in the MRXI simulation environment (see bottom panels of Figure 2a). The reason why they are not modeled in the virtual representations of the cylindrical coil configurations is because the FCPs are thin enough that the resulting magnetic field of the connection traces can be neglected. Nonetheless, the bending of the coils around the tubular phantom container is considered in the models, which is visible in the bottom panels of Figure 2b.

The CC values in Table 2 prove that every phantom in both ROIs is reconstructed more accurately when using the optimized instead of the regular coil configurations, either by SWTikh or by SWISTA. Also, the comparison of the CC values among the two individual regularization methods shows that the optimized coil configurations consistently yield more accurate reconstructions. These results validate the conclusions drawn from the simulation study^35^ and confirm the hypothesis that MRXI setups with system matrices optimized with respect to their condition number κ(L) are better suited for the reconstruction of arbitrary MNP distributions than intuitive coil configuration designs.

Due to the sparsity of the employed MNP phantoms, one would assume that SWISTA should be better suited for their reconstruction than SWTikh. Nonetheless, SWTikh yields higher CC values in many cases which are attributed to particular numerical properties of L. Efficient $\ell_{1}$‐regularization (i.e., SWISTA) necessitates certain system matrix properties, in particular uncorrelated columns,^44^ which are not given in MRXI.^28^ Hence, the sparsity of the phantoms cannot be fully exploited by the SWISTA algorithm, leading to reconstructions with imaging qualities similar to SWTikh.

It should be noted that slightly better reconstructions (at best <2% increase of the CC values) are enabled by lowering the termination criterion of the regularization algorithms, thus allowing for increased numbers of iterations. However, due to the marginal improvements and the considerably increased runtimes (>1min per reconstruction instead of few seconds), the termination criterion was fixed at 0.1% minimum deviation, as described in Section 2.5.

The signal quality measures as summarized in Table 2 indicate that the optimized coil configurations are generally more likely to yield good measurement data. This can be attributed to the fact that the regular coil configurations exhibit consistent coil radii, whereas the optimized coils employed here are typically larger beneath the ROIs and smaller on top, where they are closer to the sensor system. Large magnetic fields occasionally induce eddy currents inside the dewar that shields the SQUID sensors which hampers accurate relaxation measurements. The smaller coils of the optimized coil configurations that lie in the vicinity of the sensors have fewer windings and produce weaker magnetic fields when using constant coil currents and thus are less disruptive for the SQUIDs. Specifically, this is evident in the comparably low D values of the measurements with the cylindrical regular coil configurations. Their large coil diameters of 3 cm produce relatively strong magnetic fields close to the sensors and thus are more detrimental to the measurement data. In contrast, the coils of the cuboidal regular arrangement exhibit smaller diameters of 2 cm, thereby causing fewer faulty recordings due to their weaker magnetic fields and exhibiting D values similar to the measurements with the optimized coil configurations. Nonetheless, even after adjusting the sensor data such that the measurements with regular and optimized coil configurations exhibit similar D values and identical amounts of usable data points $N_{\mathrm{usable}}$, the optimized configurations consistently yield more accurate imaging results and on average a more precise quantification of the MNP iron masses as is evident in Table 3.

Even though the reconstruction performance of MRXI was successfully improved with our optimization, some attention should be given to the remaining aliasing artifacts. Due to the low‐frequency spatial encoding of the ROI during MRXI, MNP phantoms containing high spatial frequencies such as the Separation phantoms or the bifurcation in the Vessel phantom are still challenging to reconstruct. As an example, this can be observed in the reconstructions of the optimized cylindrical MRXI setup in Figure 7, where MNPs are in part incorrectly recovered in voxels adjacent to their true sources. This becomes more problematic with increasing distances between the voxels and the coils/sensors. Thus, it is important to assess the source separation capabilities of an MRXI setup prior to its (clinical) application.

The present study provides a proof of concept that the optimization of MRXI coil configurations with respect to the condition number of the system matrix is indeed expedient for maximizing the imaging quality in practical applications. It was possible to reduce the condition numbers of the optimized configurations by roughly one order of magnitude compared to the κ(L) values of the regular configurations. However, the adapted coil positions and radii as suggested here represent only a subset of the available design parameters that ultimately can be optimized in MRXI setups. In this respect, it was already shown in simulations that the optimization of the excitation coil currents,^28^ coil orientations,^45^ and coil shapes^33^ result in increased MRXI imaging qualities. Additionally, other parameters such as the sensor positions and orientations have not been considered yet in an MRXI optimization approach. Since all of these individual design parameters influence the MRXI reconstruction accuracy to some degree, it is reasonable to assume that the joint optimization of all those variables would result in substantially improved imaging quality. Van Durme et al. demonstrated the magnitude of such a joint optimization based on a tomographic imaging setup for MNPs related to MRXI.^46^ Multiple design parameters (drive coil radii and positions, number of coil turns, and the frequencies of the applied magnetic fields) were optimized with respect to κ(L) in the course of their simulation study which resulted in far more accurate reconstructions than previously obtained from their prototype configuration of the imaging setup. Therefore, the joint optimization of multiple MRXI design variables is still an important step for the clinical applicability of this imaging modality. The technical feasibility of such an approach has been proven in the present study by optimizing the MRXI excitation coil configurations. Consequently, one of the next steps for advancing MRXI should involve the practical realization of a multiparameter optimized imaging setup (e.g., coils, sensors, as well as excitation coil currents).

## 5 CONCLUSION AND OUTLOOK

In this study, we optimized the coil positions and radii of two distinct MRXI setups by minimizing the respective system matrix condition numbers in order to maximize reconstruction quality. In an experimental comparison, these configurations outperformed two regular coil grids with respect to imaging quality. The reconstructions of a total of seven different magnetic nanoparticle phantoms were superior for the optimized coil configurations both qualitatively as well as quantitatively in almost every case. Thus, we were able to demonstrate for the first time in practice the technical feasibility and efficacy of optimizing MRXI setups by minimizing their system matrix condition numbers. Based on the promising results shown in our study, we infer that the minimization of the system matrix condition number provides an elegant way to optimize not only the coil geometries but also other design parameters of magnetorelaxometry imaging setups (e.g., sensor configuration, coil currents, etc.). This will enhance the achievable reconstruction qualities even further and will eventually assist in developing future clinical MRXI scanners.

The envisioned upscaling of MRXI to enable MNP imaging inside large ROIs, like a torso or the full body, is accompanied by increasing voxel distances to coils and sensors that inevitably lead to more ill‐posed inverse problems when maintaining or even decreasing the voxel sizes used in smaller setups. Naturally, coarser voxel grids can be used to mitigate the ill‐posedness, but finer resolutions will require more sophisticated spatial encoding schemes, for instance, optimized magnetic field configurations as shown here and in our earlier work,^28^ fully exploiting the temporal information in the relaxation signals,^47^ more flexible sensor positioning using the novel OPM technology,^26^ and likely additional strategies. Nevertheless, the upscaling of the modality while enabling clinically relevant spatial resolution remains a challenging task and will positively be one focus of MRXI in the near future.

Even though several issues still need to be addressed (e.g., human‐induced movement and breathing artifacts and changing relaxation characteristics within the body), this technology could improve (early) cancer detection methods,^16^, ^34^ and facilitate quantitative MNP imaging for magnetic drug targeting^1^, ^2^ and magnetic hyperthermia^3^, ^4^, ^5^ inside large body regions. The investigation of selected organs/tumor regions like the brain, prostate, liver, or breast should be directly possible after adapting the excitation coil system. Here, a quantitative determination and monitoring of the MNP distribution would be promising from a clinical medical imaging or diagnostics perspective. Since for these scenarios, the same or only slightly enlarged extensions of ROI would be used, we would roughly estimate the same spatial and temporal resolution as well as sensitivity as in the present state of MRXI.

## CONFLICT OF INTEREST

The authors have no relevant conflicts of interest to disclose.
